# Recombinant *Porphyromonas gingivalis* W83 FimA alters immune response and metabolic gene expression in oral squamous carcinoma cells

**DOI:** 10.1002/cre2.588

**Published:** 2022-05-15

**Authors:** Sabine E. Groeger, Martina Hudel, Silke Zechel‐Gran, Jens M. Herrmann, Trinad Chakraborty, Eugen Domann, Joerg Meyle

**Affiliations:** ^1^ Department of Periodontology Justus‐Liebig University of Giessen Giessen Germany; ^2^ Institute of Medical Microbiology Justus‐Liebig University of Giessen Giessen Germany; ^3^ German Center for Infection Research (DZIF) Partner Site Giessen‐Marburg‐Langen Giessen Germany; ^4^ Institute of Hygiene and Environmental Medicine Justus‐Liebig University of Giessen Giessen Germany

**Keywords:** oral carcinoma cells, *P. gingivalis*, recombinant FimA, transcriptomics

## Abstract

**Objectives:**

The Gram‐negative anaerobic rod *Porphyromonas gingivalis* (*P. gingivalis*) is regarded as a keystone pathogen in periodontitis and expresses a multitude of virulence factors iincluding fimbriae that are enabling adherence to and invasion in cells and tissues. The progression of periodontitis is a consequence of the interaction between the host immune response and periodontal pathogens. The aim of this study was to investigate the genome‐wide impact of recombinant fimbrial protein FimA from *P. gingivalis* W83 on the gene expression of oral squamous carcinoma cells by transcriptome analysis.

**Materials and Methods:**

Human squamous cell carcinoma cells (SCC‐25) were stimulated for 4 and 24 h with recombinant FimA. RNA sequencing was performed and differential gene expression and enrichment were analyzed using gene ontology (GO), Kyoto Encyclopedia of Genes and Genomes (KEGG), and REACTOME. The results of transcriptome analysis were validated using quantitative real‐time polymerase chain reaction (PCR) with selected genes.

**Results:**

Differential gene expression after 4 and 24 h revealed upregulation of 464 (4 h) and 179 genes (24 h) and downregulation of 69 (4 h) and 312 (24 h) genes. GO, KEGG, and REACTONE enrichment analysis identified a strong immunologic transcriptomic response signature after 4 h. After 24 h, mainly those genes were regulated, which belonged to cell metabolic pathways and replication. Real‐time PCR of selected genes belonging to immune response and signaling demonstrated strong upregulation of CCL20, TNFAIP6, CXCL8, TNFAIP3, and NFkBIA after both stimulation times.

**Conclusions:**

These data shed light on the RNA transcriptome of human oral squamous carcinoma epithelial cells following stimulation with *P. gingivalis* FimA and identify a strong immunological gene expression response to this virulence factor. The data provide a base for future studies of molecular and cellular interactions between *P. gingivalis* and oral epithelium to elucidate basic mechanisms that may provide new prospects for periodontitis therapy and give new insights into the development and possible treatments of cancer.

## 1 INTRODUCTION

Periodontitis is a chronic inflammatory disease that impairs not only the sulcular epithelium and its connective tissue but also the supporting structures of the teeth such as the periodontal ligament and the alveolar bone (Armitage, 2004; Nakano et al., 2008).

One component that supports the inflammatory process is the formation and persistence of a microbial biofilm in the subgingival environment and on the tooth surface. This biofilm triggers the host's immune response and stimulates the monocytic production and release of proinflammatory cytokines and lysosomal enzymes (Socransky & Haffajee, 2002).

Furthermore, fibroblasts react with reduced collagen synthesis and the connective tissue responds with increased matrix‐metalloproteinase production together with accompanying reduction of their inhibitors. This disbalance initiates the destruction of periodontal tissue and bone resorption that is caused by the chronicity of the microbial and inflammatory affection (Sbordone & Bortolaia, 2003).

Chronic infections are known to be associated with carcinoma development and progression.

Oral cancers are the world's 11th most common human neoplasm and account for 3% of all newly diagnosed cancer cases (Parkin et al., 2005; Reid et al., 2000). More than 90% are oral squamous cell carcinomas (OSCC; Funk et al., 2002; Muir & Weiland, 1995).

It was shown that chronic periodontitis increases the risk for tongue and head and neck carcinomas (Tezal et al., 2009, 2007).

Among the diverse microorganisms that have been identified in the pathogenesis of periodontitis, the Gram‐negative anaerobic rod *Porphyromonas gingivalis* (*P. gingivalis*) is regarded as a keystone pathogen (Hajishengallis et al., 2012). Strain dependent, it expresses a variety of virulence factors such as a capsule, proteins of the outer membrane, lipopolysaccharide (LPS), proteases like gingipains, collagenases, hemolysin, trypsin proteases, hemagglutinins, and fimbriae (Amano et al., 1999; Holt et al., 1999; Inaba et al., 2008). The fimbriae are essential for colonization, invasion, establishment, and persistence within the host. Furthermore, they play a role in immune evasion by destructing its mechanisms and supporting damage to the protective periodontal tissues (Amano et al., 1999; Holt et al., 1999). The fimbriae of *P. gingivalis* are considered as main virulence factor among the virulence factors that this germ is able to express. This consideration is due to its capability to adhere to and invade cells and tissues, which explains its pathogenicity, especially in periodontal tissues (Nakagawa et al., 2002).

Periodontal pathogens play an essential role in the initiation of periodontitis as they can directly destruct periodontal tissues through the release of toxic factors and metabolites. Nevertheless, the progression of periodontitis is maintained mostly by the interaction between the host immune response and the periodontal pathogens. The damage caused by periodontal pathogens is persistent since it results in long‐lasting periodontal tissue damage (Meyle et al., 2017). It is known that a bacterium primarily activates innate immune responses to combat pathogenic challenges, which also is a necessary step for the subsequent adaptive immunity. Typical pathogenic molecular patterns are recognized by a multitude of cell surface receptors, the so‐called pattern recognition receptors, including toll‐like receptors (TLRs) and NOD‐like receptors (NLRs). After binding the pathogen‐associated molecular patterns by these receptors, the intracellular signal‐transmission pathways are activated. This process stimulates the expression of numerous inducible costimulators and leads to the release of inflammatory and chemotactic factors (Akira et al., 2006).

Among the multiple chemokines, the CC chemokine receptor–ligand pair, CC chemokine receptor 6 (CCR6), and CC chemokine ligand 20 (CCL20), play an important role in immunological research because of their therapeutic potential. CCL20 is a small cytokine that is a member of the CC chemokine family. It is strongly chemotactic for lymphocytes and can attract neutrophils (Baba et al., 1997). CCR6, its binding partner, belongs to the G protein‐coupled receptor superfamily and is expressed on immature dendritic cells (DCs), a number of T‐cells (Th1, Th2, Th17, Treg), B‐cells, natural killer cells (NKT cells), and also on neutrophils (Schutyser et al., 2003). The properties of CCR6 and CCL20 show their functions to coordinate both immune homeostasis and immune activation. It has been established that the immunological importance of this chemokine‐receptor–ligand union affects human health and disease with extensive consequences including multiple organs of the body. A multitude of research studies showed that the CCR6 and CCL20 axis has a direct impact on multiple systems, such as the gastrointestinal, excretory, respiratory, nervous, reproductive, and skeletal system. It influences the different systems by various immune mechanisms. The CCL20–CCR6 receptor–ligand pair provides a promising therapeutic target because of its major relevance in clinical pathophysiology. Blockade or inhibition of either partner potentially constitutes successful pharmacotherapy as a treatment for related diseases (Ito et al., 2011; Proudfoot, 2002; Ranasinghe & Eri, 2018).

Tumor necrosis factor‐inducible gene 6 (TNFAIP6) encodes tumor‐necrosis factor (TNF)‐stimulated gene/protein 6 (TSG‐6), which is an inflammation‐associated protein, has been demonstrated to be upregulated by proinflammatory mediators, such as interleukin‐1, TNF, and LPS in a number of cells and in the context of inflammation and inflammatory diseases (Bayliss et al., 2001; Lee et al., 2009; Milner et al., 2006).

TFNAIP6 was initially detected at high levels in the joints of patients suffering from rheumatoid and osteoarthritis, which suggested a proinflammatory role (Wisniewski et al., 1993). In contrast, the application of TSG‐6 caused inhibited damage in some inflammatory models such as arthritis (Glant et al., 2002; Mindrescu et al., 2002), suggesting that it induces anti‐inflammatory effects. TSG‐6 has also been found as the main mediator of anti‐inflammatory properties of human mesenchymal stem cells in diverse models, including myocardial infarction (Lee et al., 2009), peritonitis (Choi et al., 2011), wound healing (Qi et al., 2014), and type 1 diabetes (Kota et al., 2013). One mechanism underlying its protective effects is thought to be its ability to inhibit the influx of neutrophils to inflammatory sites and the concomitant neutrophil‐induced damage (Danchuk et al., 2011; Lee et al., 2009; Szanto et al., 2004).

The aim of this study was to investigate the genome‐wide impact of recombinant FimA from *P. gingivalis* W83 on the gene expression of oral squamous carcinoma cells by transcriptome analysis and to verify the results by quantitative real‐time polymerase chain reaction (qRT‐PCR) of selected genes.

## 2 MATERIALS AND METHODS

### 2.1 Recombinant FimA

The recombinant FimA protein was prepared as described elsewhere (Groeger et al., 2021).

Briefly, the FimA‐gene was amplified by PCR, purified, cloned in the vector, and transferred into *Listeria innocua* (*L. innocua*). The supernatant of the grown bacteria was harvested and the expressed protein was purified using fast protein liquid chromatography via a His trap HP column. The protein was prepared and characterized by lauryl sulfate‐polyacrylamide gel electrophoresis and mass spectrometry.

### 2.2 Cell culture

The human oral tongue squamous cell carcinoma cell line SCC‐25 was purchased from the DSMZ (German Collection of Microorganisms and Cell Cultures, Braunschweig, Germany, DSMZ numbers ACC 617). Cells were cultured in a medium containing Dulbecco's minimal essential medium:Ham's F12 (4:1 vol/vol), Hepes buffer (Invitrogen, Karlsruhe, Germany), and 10% fetal calf serum (Greiner, Frickenhausen, Germany). The cells were seeded in six‐well plates at 1 × 10^6^ cells per well and grown at 37°C in a humidified atmosphere with 5% CO_2_ to 80% confluency before stimulation. The cells were stimulated using 10 µg/ml recombinant FimA protein for 4 and 24 h.

### 2.3 RNA extraction

After incubation, cells were lysed and the RNA was extracted using NucleoSpin® RNA Plus (Machery‐Nagel, Munic, Germany) columns following the manufacturer's instructions. The concentration and quality of the RNA were measured using a NanoDrop microvolume spectrophotometer (Thermo Fisher Scientific, Dreieich, Deutschland).

### 2.4 RNA‐seq

After quality control, messenger RNA (mRNA) from eukaryotic organisms was enriched using oligo(dT), fragmented randomly in fragmentation buffer, followed by complementary DNA (cDNA) synthesis using random hexamers and reverse transcriptase. After first‐strand synthesis, the second strand was synthesized by nick‐translation. The final cDNA library is ready after a round of purification, terminal repair, A‐tailing, ligation of sequencing adapters, size selection, and PCR enrichment. Library concentration was first quantified and then sequencing was done using an Illumina device. After obtaining the results, analysis of differential expression analysis was performed. Cluster analysis was performed to find genes with similar expression patterns under various experimental conditions. Enrichment analysis of the differential expressed genes was done to find out which biological functions or pathways were significantly associated with differentially expressed genes. Gene ontology (GO, http://www.geneontology.org/) enrichment analysis was the next step. GO enrichment analysis is used by GOseq, which is based on Wallenius noncentral hypergeometric distribution. Kyoto Encyclopedia of Genes and Genomes (KEGGs) enrichment analysis was performed to find out interactions of multiple genes that may be involved in certain biological functions, such as enriched metabolic pathways or signal transduction pathways, associated with differentially expressed genes compared with the whole genome background. A further instrument for the interpretation of the results is an analysis using REACTOME, a peer‐reviewed bioinformatics database for biological processes and pathways.

### 2.5 Quantitative real‐time PCR

Expression of mRNA was assayed 4 and 24 h after infection. The cDNA synthesis was performed with the Verso™ cDNA Kit (Thermo Fisher Scientific, Dreieich, Deutschland). A qRT‐PCR was performed with the SensiFast no ROX SYBR Green Mix according to the manufacturer's instructions (Bioline, Luckenwalde, Germany). The following primers were used: QuantiTect Primer Assay (Qiagen, Hilden, Germany) Hs_CCL20_1_SG (CCCL20), Hs_CXCL8_1_SG (CXCL8), Hs_TNFAIP3_1_SG TNFAIP3), Hs_NFκBIA_1_SG (NFκBIA), Hs_TNFAIP6_1_SG as target genes and Hs_GAPDH_1‐SG (GAPDH) as housekeeping gene (patents; Roche Molecular Systems, Roche Diagnostics International AG, Rotkreuz, Switzerland). Cycling and detection was performed in a Bio‐Rad CX96 cycler (Bio‐Rad, Feldkirchen, Germany). The values were analyzed using the comparative CT (ΔΔ*C*_t_) method. The amount of target ($2^{‐∆∆C_{t}}$) was obtained by normalizing an endogenous reference (GAPDH) relative to noninfected cells.

### 2.6 Statistical analysis

All investigations were performed in three different independent experiments. The results were analyzed using an independent two‐sample Student's *t*‐test. The character of the evaluation was explorative. Probability of error was set to 5% and shown as *p* values, *n* = 3, *p < .05, ^‡^ *p* < .01.

### 2.7 Ethical considerations

All experiments followed the guidelines of good clinical/laboratory practise (GCP/GLP) and the WHO declaration, Helsinki 1964, latest update Seoul 2008 (59th WMA General Assembly, Seoul, October 2008).

## 3 RESULTS

The response of human oral squamous carcinoma cells (SCC‐25) to stimulation with recombinant FimA was assessed by RNA‐seq of total RNA extracted from SCC‐25 cells.

**Figure 1:**
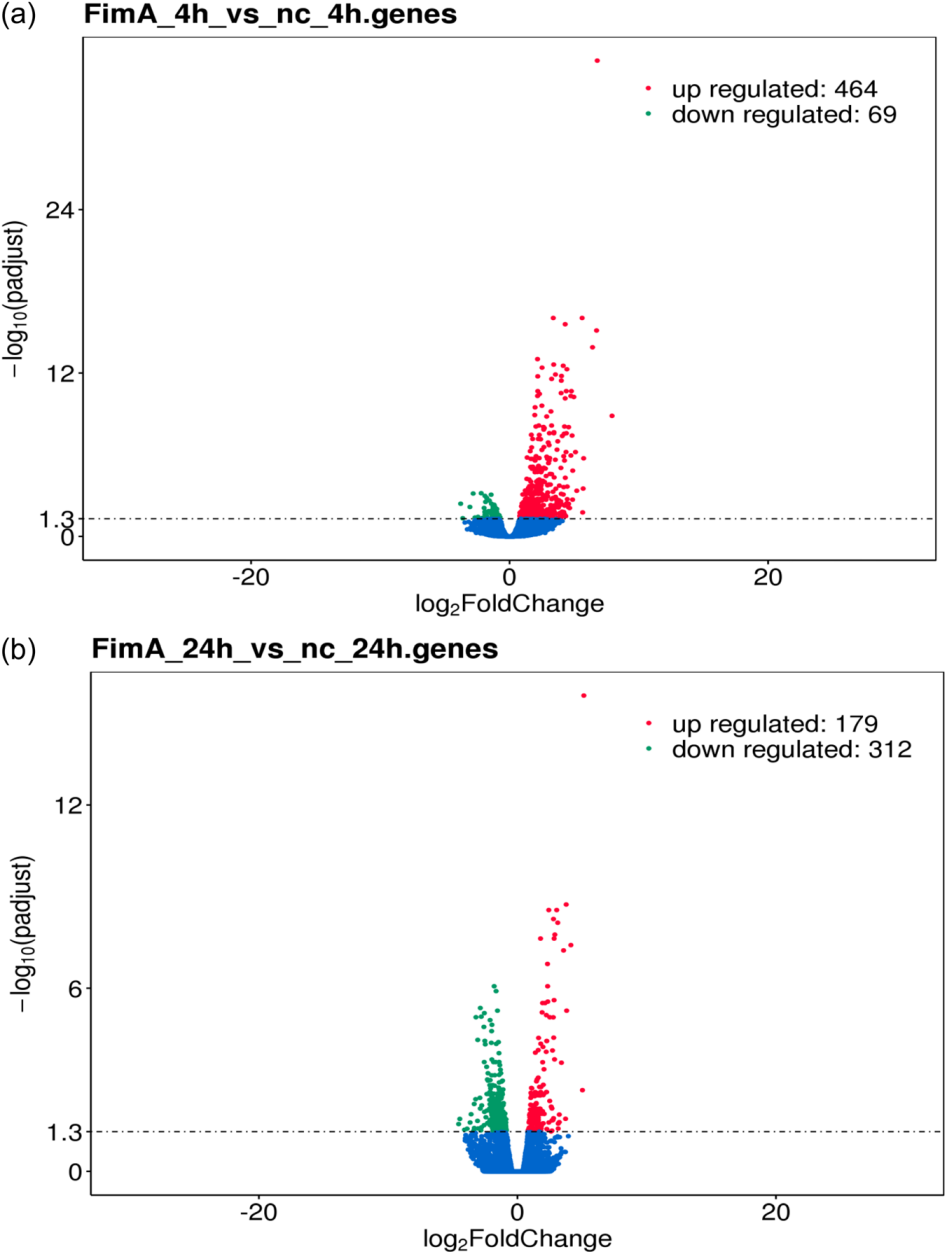
Differential expression analysis of SCC‐25 cells stimulated with FimA (compared to the nonstimulated negative control. The input data for differential gene expression analysis is read counts from gene expression level analysis. The differential gene expression analysis contains read counts normalization, model‐dependent *p*‐value estimation, and FDR value estimation based on multiple hypothesis testing. The results are shown as volcano plots; the changes are indicated as log 2 fold change, *p* adjust is the normalized *p*‐value. (a) Stimulation for 4 h, (b) stimulation for 24 h. FDR, false discovery rate; FimA, FimA stimulated cells; NC, nonstimulated cells as negative control; SCC, squamous cell carcinoma cell.

**Figure 2:**
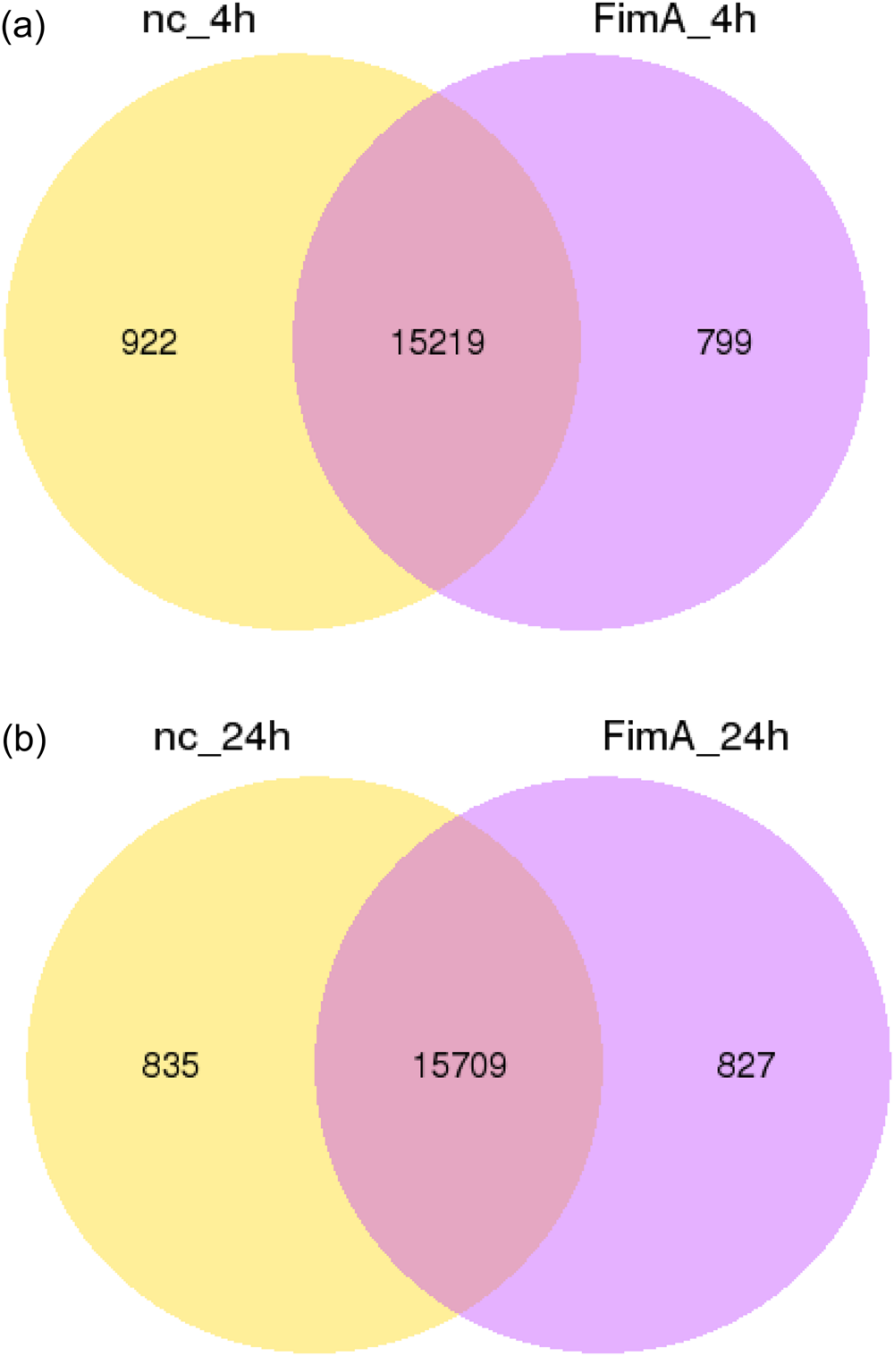
Coexpression of genes are shown as Venn diagrams. (a) Stimulation for 4 h, (b) stimulation for 24 h. FimA, FimA‐stimulated cells; NC, nonstimulated cells (negative control).

Differential expression analysis after 4 h FimA stimulation compared to the negative control demonstrated upregulation of 464 (4 h; Figure 1a) and 179 (24 h; Figure 1b) genes and downregulation of 69 (24 h; Figure 1a) and 321 (24 h; Figure 1b). After the 24 h stimulation 179 genes were upregulated and 312 downregulated Figure 1b. Coexpression analysis revealed that after 4 h 15,219 genes were coexpressed, 922 genes were differentially expressed by the negative control, and 799 by the FimA stimulated cells (Figure 2a). The 24 h stimulation showed 15,709 coexpressed genes, 835 differentially expressed by the negative control, and 827 by the stimulated cells (Figure 2b).

**Figure 3:**
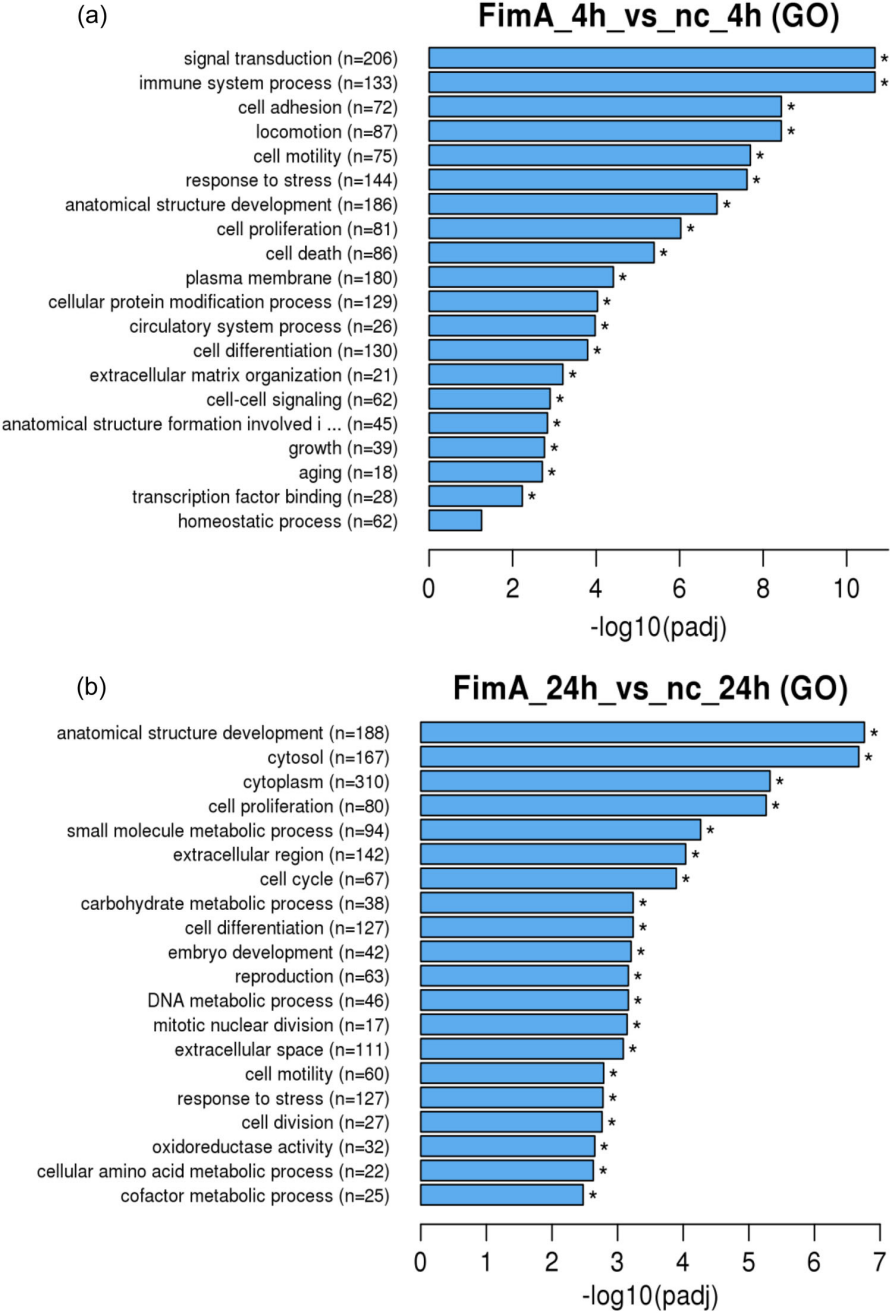
Enrichment analysis using gene ontology (GO) of the differentially expressed genes by SCC‐25 cells is demonstrated in a directed acyclic graph structure. (a) 4 h Stimulation with recombinant FimA, (b) 24 h stimulation with FimA. In general, GO terms with corrected *p* < .05 are significant enrichment. *p*_adj_, adjusted *p*‐value; SCC, squamous cell carcinoma cell.

Genes that are associated with distinct biological processes were clustered. GO analysis (Figure 3) revealed that after 4 h (Figure 3a) mostly those genes were upregulated, which are involved in biological processes, such as signal transduction (*n* = 206), immune system processes (*n* = 133), response to stress (*n* = 144), cell motility (*n* = 75), locomotion (*n* = 87), cell adhesion (*n* = 71), anatomical structure development (*n* = 186), cell proliferation (*n* = 81), cell death (*n* = 86), cellular protein modification process (*n* = 129), cell differentiation (*n* = 130), and cell–cell signaling (*n* = 62).

**Figure 4:**
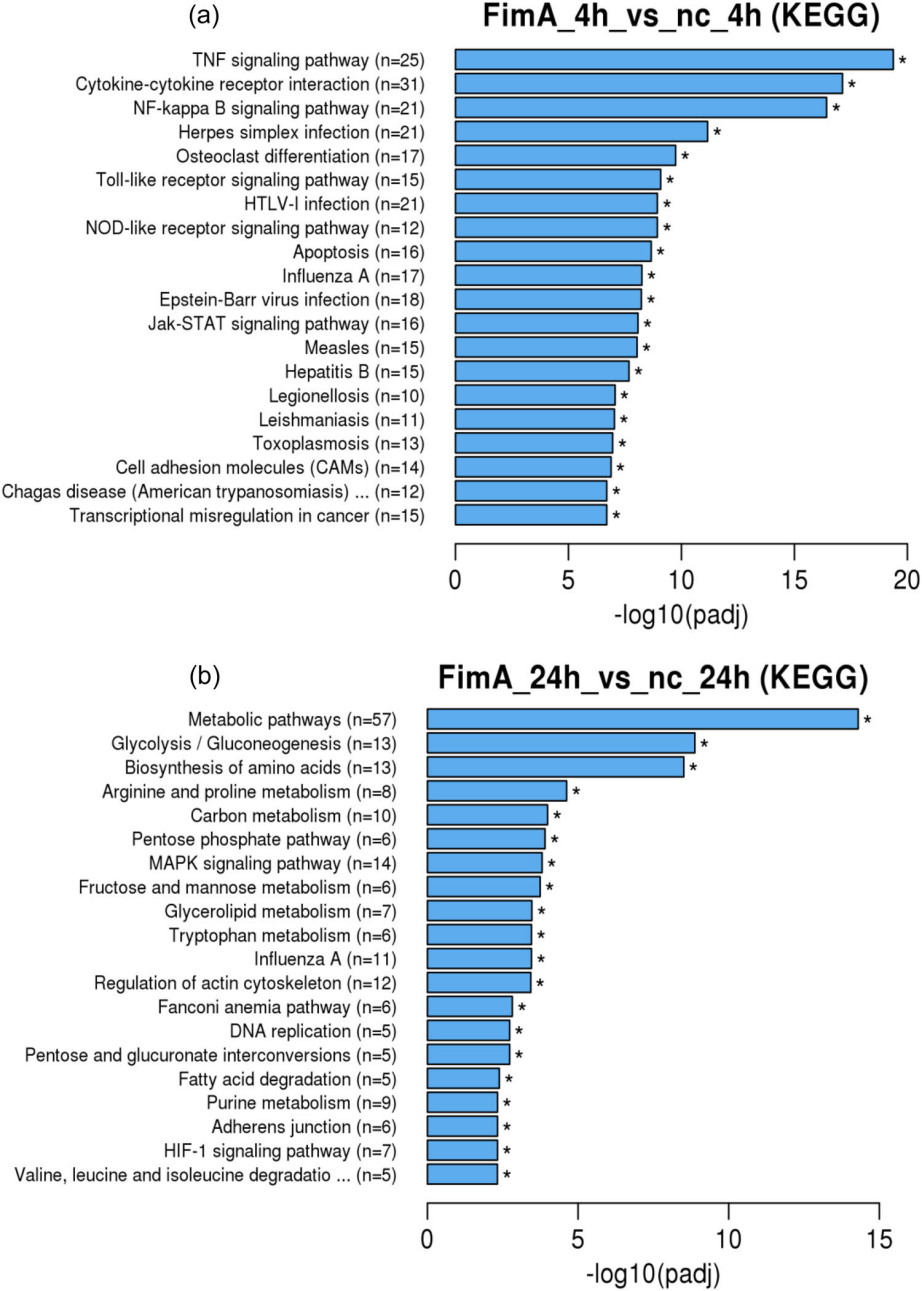
Kyoto Encyclopedia of Genes and Genomes (KEGG) analysis of the differentially expressed genes by SCC‐25 cells. Gene expression of pathways and signal transduction are demonstrated in the graph structure. (a) 4 h, (b) 24 h stimulation with FimA. Generally, GO terms with corrected *p* < .05 are significant enrichment. GO, gene ontology; *p*_adj_, adjusted *p*‐value; SCC, squamous carcinoma cell.

After 24 h (Figure 3b) upregulation mainly consisted of genes that are associated to response to stress (*n* = 127), cell motility (*n* = 60), locomotion (*n* = 65), small molecule metabolic process (*n* = 94), anatomical structure development (*n* = 188), cell proliferation (*n* = 80), cell cycle (*n* = 67), DNA metabolic process (*n* = 46), cell differentiation (*n* = 127), and cell division (*n* = 27). KEGG analysis (Figure 4) was also performed. After 4 h of stimulation with FimA (Figure 4a), genes were upregulated and were related to the following biological functions: Cytokine‐receptor interaction (*n* = 31), NLR signaling pathway (*n* = 12), TLR signaling pathway (*n* = 21), NF‐kappa B signaling pathway (*n* = 21), TNF signaling pathway (*n* = 25), TLR signaling pathway (*n* = 15), apoptosis (*n* = 16), Janus kinase–signal transducer and activator of transcription signaling pathway (*n* = 16), and osteoclast differentiation (*n* = 17). After 24 h (Figure 4b), genes that were upregulated were found in the following biological functions: metabolic pathway (*n* = 57), glycolysis/gluconeogenesis (*n* = 13), biosynthesis of amino acids (*n* = 13), mitogen‐activated protein kinase (MAPK) signaling pathway (*n* = 14), DNA replication (*n* = 5), adherens junction (*n* = 6), and hypoxia‐inducible factor 1 signaling pathway (*n* = 7).

**Figure 5:**
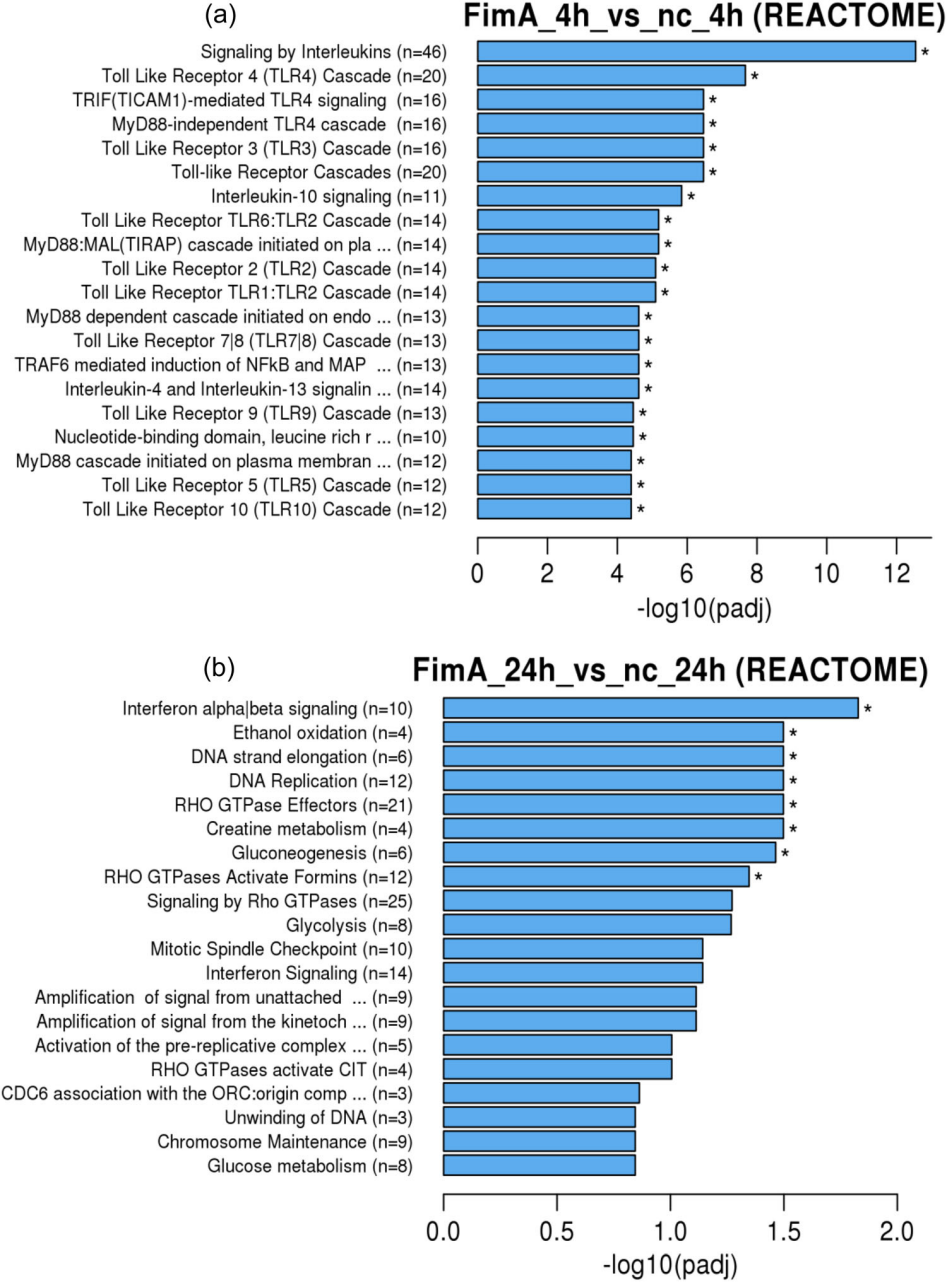
REACTOME analysis of the differentially expressed genes by SCC‐25 cells. Gene expression of pathways and signal transduction are shown as graphics. (A) 4 h Stimulation with recombinant FimA, (b) 24 h stimulation with FimA. Generally, GO terms with corrected *p* < .05 significant enrichment. GO, gene ontology; *p*_adj_, adjusted *p*‐value; SCC, squamous carcinoma cell.

The analysis using a peer‐reviewed bioinformatics database for biological processes and pathways = REACTOME (Figure 5) showed that compared to the negative control genes related to the following biological processes were upregulated after 4 h of incubation (Figure 5a) with Fim: toll‐like receptor 10 cascade (*n* = 12), toll‐like receptor 5 cascade (*n* = 12), MyD88 cascade initiated on plasma membrane (*n* = 12), nucleotide‐binding domain (*n* = 10), toll‐like receptor 9 (TLR9) cascade (*n* = 13), interleukin‐4 and interleukin‐13 signaling (*n* = 14), TNF receptor‐associated factor 6 mediated induction of nuclear factor‐kappa B (NF‐κB) and MAPK (*n* = 13), toll‐like receptor 7|8 cascade (*n* = 13), MyD88 dependent cascade (*n* = 13), toll‐like receptor TLR1:TLR2 cascade (*n* = 14) toll‐like receptor 2 (TLR2) cascade (*n* = 14), myeloid differentiation primary response (MyD88:) MyD88 adapter‐like (MAL)(toll/interleukin‐1 receptor domain‐containing adapter protein TIRAP) cascade (*n* = 14), toll‐like receptor TLR6:TLR2 cascade (*n* = 14), interleukin‐10 signaling (*n* = 11) TLR cascades (*n* = 20), toll‐like receptor 3 (TLR3) cascade (*n* = 16), MyD88‐independent toll‐like receptor 4 (TLR4) cascade (*n* = 16), TIR‐domain‐containing adapter‐inducing interferon‐β (TRIF) (TIR domain‐containing adapter molecule 1 = TICAM1)−mediated TLR4 signaling (*n* = 16), and TLR4 cascade (*n* = 20). After 24 h (Figure 5b) genes for interferon alpha/beta signaling (*n* = 14), chromosome maintenance (*n* = 9), unwinding of DNA (*n* = 3), cell division cycle 6 association with the origin recognition complex (*n* = 3), RHO GTPases activate CIT (*n* = 4), activation of the prereplicative complex (*n* = 5) mitotic spindle checkpoint (*n* = 10), glycolysis (*n* = 8), signaling by Ras homolog (Rho) guanosine triphosphatases (GTPases) (*n* = 25), RHO GTPases activate formins (*n* = 12), gluconeogenesis (*n* = 6), creatine metabolism (*n* = 4), RHO GTPase effectors (*n* = 21), and DNA replication (*n* = 12) were regulated.

**Figure 6:**
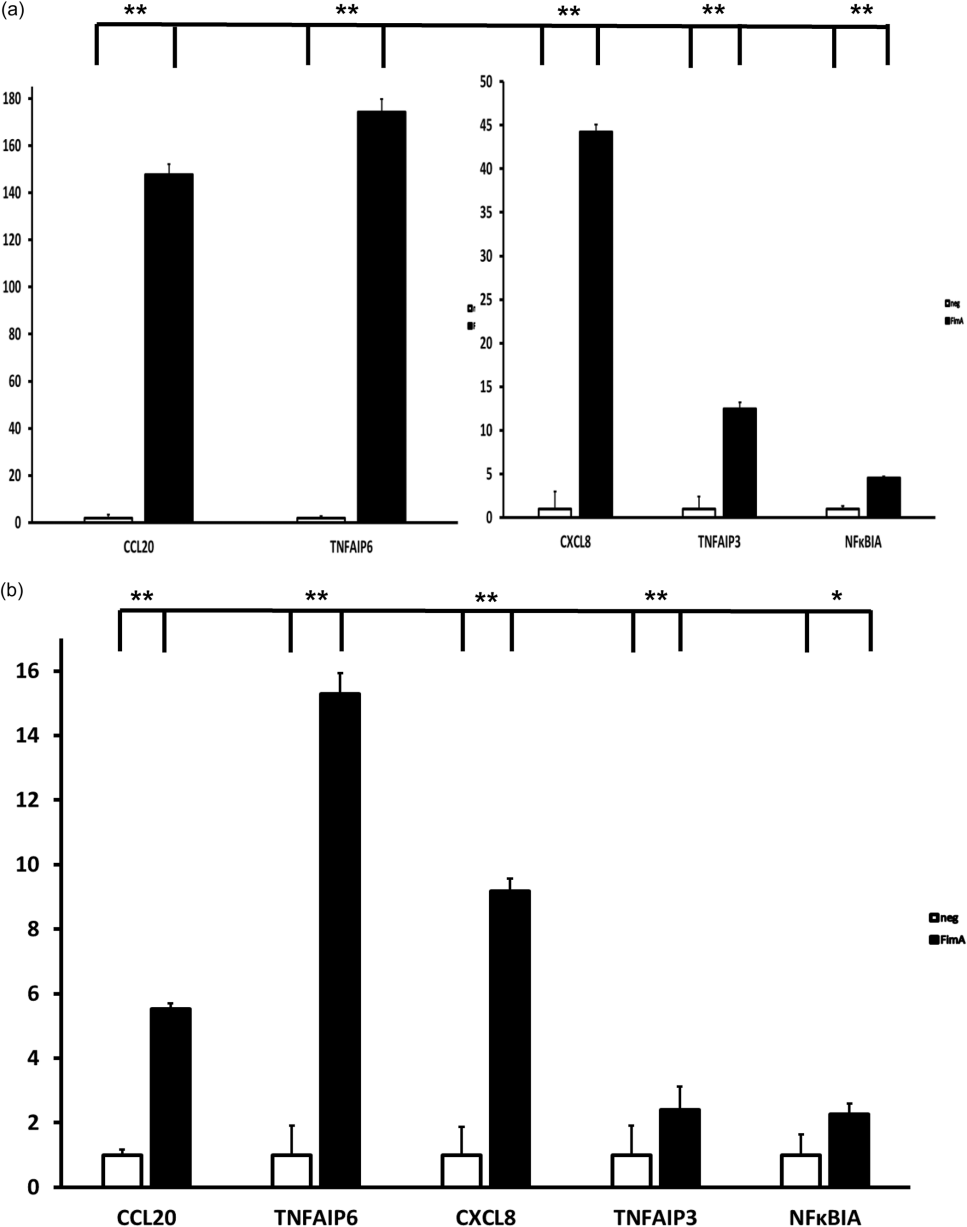
Validation of the results of the RNA‐seq using quantitative real‐time PCR with five chosen genes analyzed by the ΔΔ*C*_t_ method, fold induction relative to nonstimulated, normalized to GAPDH, *p < .05, **p < .01 (*n* = 3). Analysis of gene expression for CCL20, TNFAIP6, CXCL8, TNFAIP3, NFKBIA. CCL20, chemokine (C‐C motif) ligand 20; CXCL8, chemokine (C‐X‐C motif) ligand 8; NFKBIA, nuclear factor‐kappa B inhibitor alpha; PCR, polymerase chain reaction; TNFAIP6, tumor necrosis factor alpha‐inducible gene 6 protein.

The results from the qPCR (Figure 6) demonstrated that the chosen genes were upregulated after stimulation with FimA in SCC‐25 cells. After 4 h (Figure 6a), a 148.6‐fold upregulation of CCL20, 174.3‐fold of TNFAIP6, 44.2‐fold of chemokine C‐X‐C motif (CXCL) 8, 12.5‐fold of TNFAIP3 and 4.6‐fold of NF‐κB inhibitor alpha (NFκBIA) and was detected. After 24 h (Figure 6b) upregulation of CCL20 was 5.5‐fold, TNFAIP6 15.3‐fold, CXCL8 9.2‐fold, TNFAIP3 2.4‐fold, and NFκBIA 2.2‐fold. Hence, all the chosen genes were upregulated and the results confirmed the results that were obtained from the transcriptome analysis.

## 4 DISCUSSION

The main factor of the pathogenicity of *P. gingivalis* in chronic periodontitis and its influence on immune response and regulation has been strongly linked to fimbriae expression. Fimbriae initiate and mediate interactions with host cells, which are a prerequisite for the initiation and progression of inflammatory periodontal diseases (Lamont & Jenkinson, 1998). Chronic inflammation is regarded as the main risk factor for cancer. Studies demonstrated that around 15% of tumors worldwide are associated with microbial infection (Kuper et al., 2000). It has been shown that increased bacterial load in periodontitis is associated with head and neck carcinoma and increases the risk for oral cancer (Tezal et al., 2009, 2007). Until today, complete expression profiles of oral carcinoma cells in response to FimA have not been described.

In this study, RNA‐seq was used to assess the complete transcriptome that is induced in oral squamous carcinoma cells after stimulation with a recombinant *P. gingivalis* W83 FimA protein and revealed that FimA challenge of the oral carcinoma cells induced upregulation of a wide array of the immune response‐related genes.

RNA‐seq is regarded as a powerful digital instrument for the measurement of gene expression and is able to sensitively analyze the transcriptomes in an unbiased and comprehensive manner (Wilhelm et al., 2008). This study presents a transcriptomic view of oral carcinoma cell response to stimulation with recombinant FimA. RNA‐sequencing of total RNA extracted from SCC‐25 cell isolates enabled differential expression, GO, pathway, and network enrichment analyses. This study revealed a molecular response in the total transcriptome with significantly differentially expressed genes. GO and pathway studies of differentially expressed total RNA identified a strong immune response signature, which we validated by qRT‐PCR for some of the immune response‐related protein‐coding transcripts.

The human OSCC cell total RNA transcriptome was distinctly impacted by stimulation with recombinant *P. gingivalis* FimA.

Stimulation with FimA for 4 h appeared to push the carcinoma cell toward a molecular phenotype that might promote inflammation. GO and pathway analyses of the RNA‐seq data identified activity in chemokine, cytokine, and immune‐response signaling‐related categories, and a majority of gene groups identified in the network analysis were transcriptional regulators linked to inflammation, including members of the NF‐κB family.

The genes chosen for validation of the results from the RNA‐seq all are part of immune response and modulation. CCL20 and CXCL8 are strong immune modulators that belong to the IL‐1 system, which expression was shown to be sensitive to *P. gingivalis* peptidylarginine deiminase (Aliko et al., 2018). CCL20 (MIP3a) is a chemokine for DCs that is able to recruit both, Th17 and Treg cells to inflamed sites. Previous studies demonstrated elevated CCL20 expression by epithelial cells stimulated with oral bacteria (Huynh et al., 2016; Milward et al., 2007). In periodontitis tissues, it was shown to be increased (Souto et al., 2014). Oral epithelial cells and six SCC cell lines (SCC‐9, SAS, BSC‐OF, HSC‐4, HSC, Ca9‐22) were demonstrated to express CCL20 mRNA. The found CCL20 expression in SCC was localized primarily at the epithelial parts corresponding to the spinous layer. These results suggest that CCL20 contributes to the oral immune response to bacterial infection and may be involved in the growth of SCC (Abiko et al., 2003).

A number of altered genes induced by FimA are related to the regulation of the NF‐κB pathway. NF‐κB is an activator of genes involved in both innate and acquired immune responses by binding to an interferon‐stimulated response element in their promoters including TNFAIP3 (Dang et al., 2016). TNFAIP3 has not been reported to be related to oral epithelial cell biology, but some studies suggest that it has a suppressing effect on osteoclastogenesis (Hong et al., 2016). Furthermore, it was shown that elevated TNFAIP3 in gingival tissue is related to decreased periodontitis together with TLR9 activity (Crump et al., 2017). NF‐κB is a major transcription regulator of the immune response, cell adhesion, differentiation, proliferation, and apoptosis (Baldwin, 2001). Five members (p50/p105, p65/RelA, c‐Rel, RelB, and p52/p100) in the NF‐κB family have been identified from which the dimeric form of NF‐κB1 p50/RelA is the primary form (Blank et al., 1992). In the nonactivated cell, NF‐κB is inactive in the cytoplasm linked to a sequestering inhibitory protein, IκBα, β, or γ. The most current protein of this family is the NF‐κBIA (Hayden et al., 2006). In the classical activation pathway, the phosphorylation and degradation of the inhibitory proteins lead to NF‐κB dissociation from the NF‐κB complex and translocation to the nucleus, where it may activate the transcription of a big number of genes (Gilmore, 2003).

It was demonstrated that NF‐κB1 and NF‐κBIA polymorphisms appear to conjointly contribute to the risk of colorectal cancer (Song et al., 2011). Furthermore, a study showed that the NF‐κB1 and NF‐κBIA polymorphism may have a role in lung carcinogenesis and prognosis (Oltulu et al., 2014).

TNFAIP6 protein interacts with chemokine CXCL8 and inhibits its presentation on and transport across the endothelial cells and also its capability to recruit neutrophils (Dyer et al., 2014). CXCL8 is a member of the chemotactic cytokine (chemokine) family of proteins, which play a major role in regulating cell migration. It mediates leukocyte cell recruitment through signaling by chemokine receptors on cell surfaces (Baggiolini, 1998; Luster, 1998; Sallusto & Baggiolini, 2008). It has been shown that TSG‐6 inhibits chemokine‐stimulated transendothelial migration of neutrophils by direct interaction between TSG‐6 and the glycosaminoglycan binding site of CXCL8. It was also found that TSG‐6 impairs the binding of CXCL8 to cell surface glycosaminoglycans and the transport of CXCL8 across an endothelial cell monolayer, so TSG‐6 could be identified as a CXCL8‐binding protein (Dyer et al., 2014). Using OSCC, it was shown that CXCL8 secreted by OSCC facilitates the migration of bone mesenchymal stem cells (BMSCs) to OSCC. TGF‐β secreted by BMSCs subsequently induces epithelial–mesenchymal transition  of OSCC to promote their proliferation, migration, and infiltration.

These results provide a molecular basis for BMSC recruitment into tumors, and how this process may induce tumor progression (Meng et al., 2020).

Interestingly all the genes that were chosen to confirm the results from the transcriptomics after 4 h stimulation with FimA were upregulated not only after 4 h but also after 24 h. Except for TNFAIP6, they were not shown significantly upregulated in the transcriptomics results. However, these results are not unreasonable since both methods, RNA‐seq and qRT‐PCR, show a snapshot of all the cellular processes that are active at the moment of the cell harvest. Furthermore, 24 h is a relatively long incubation period. A multitude of biological processes may pass off during this time period and can affect the cells.

In summary, these data shed light on the RNA transcriptome of human oral squamous carcinoma epithelial cells upon stimulation with *P. gingivalis* FimA and identified a strong immunological gene expression response to this virulence factor.

The data we present provide a solid base for future studies of molecular and cellular interactions between *P. gingivalis* and oral carcinomas to elucidate the basic mechanisms of periodontal disease and the development of cancer. Further studies are necessary to obtain a better understanding of the mechanisms of initiation and progression of infectious and malignant oral diseases.

## AUTHOR CONTRIBUTIONS

Sabine E. Groeger performed/analyzed the experiments and wrote the manuscript. Martina Hudel, Silke Zechel‐Gran participated in the experiments. Eugen Domann participated in the experiments and analysis. Trinad Chakraborty and Jens M. Herrmann participated in the analysis. Joerg Meyle participated in the analysis and writing and supervised the study.

## CONFLICTS OF INTEREST

The authors declare no conflicts of interest.

## Data Availability

All data generated or analyzed during this study are included in this published article.

## References

[cre2588-bib-0001] Abiko, Y. , Nishimura, M. , Kusano, K. , Nakashima, K. , Okumura, K. , Arakawa, T. , Takuma, T. , Mizoguchi, I. , & Kaku, T. (2003). Expression of MIP‐3alpha/CCL20, a macrophage inflammatory protein in oral squamous cell carcinoma. Archives of Oral Biology, 48, 171–175. 12642237 10.1016/s0003-9969(02)00167-x

[cre2588-bib-0002] Akira, S. , Uematsu, S. , & Takeuchi, O. (2006). Pathogen recognition and innate immunity. Cell, 124, 783–801. 16497588 10.1016/j.cell.2006.02.015

[cre2588-bib-0003] Aliko, A. , Kaminska, M. , Bergum, B. , Gawron, K. , Benedyk, M. , Lamont, R. J. , Malicki, S. , Delaleu, N. , Potempa, J. , & Mydel, P. (2018). Impact of *Porphyromonas gingivalis* peptidylarginine deiminase on bacterial biofilm formation, epithelial cell invasion, and epithelial cell transcriptional landscape. Scientific Reports, 8, 14144. 30237466 10.1038/s41598-018-32603-y PMC6147916

[cre2588-bib-0004] Amano, A. , Nakagawa, I. , Kataoka, K. , Morisaki, I. , & Hamada, S. (1999). Distribution of *Porphyromonas gingivalis* strains with fimA genotypes in periodontitis patients. Journal of Clinical Microbiology, 37, 1426–1430. 10203499 10.1128/jcm.37.5.1426-1430.1999 PMC84792

[cre2588-bib-0005] Armitage, G. C. (2004). Periodontal diagnoses and classification of periodontal diseases. Periodontology 2000, 34, 9–21. 14717852 10.1046/j.0906-6713.2002.003421.x

[cre2588-bib-0006] Baba, M. , Imai, T. , Nishimura, M. , Kakizaki, M. , Takagi, S. , Hieshima, K. , Nomiyama, H. , & Yoshie, O. (1997). Identification of CCR6, the specific receptor for a novel lymphocyte‐directed CC chemokine LARC. Journal of Biological Chemistry, 272, 14893–14898. 9169459 10.1074/jbc.272.23.14893

[cre2588-bib-0007] Baggiolini, M. (1998). Chemokines and leukocyte traffic. Nature, 392, 565–568. 9560152 10.1038/33340

[cre2588-bib-0008] Baldwin AS, Jr. (2001). Series introduction: The transcription factor NF‐kappaB and human disease. Journal of Clinical Investigation, 107, 3–6 11134170 10.1172/JCI11891 PMC198555

[cre2588-bib-0009] Bayliss, M. T. , Howat, S. L. , Dudhia, J. , Murphy, J. M. , Barry, F. P. , Edwards, J. C. , & Day, A. J. (2001). Up‐regulation and differential expression of the hyaluronan‐binding protein TSG‐6 in cartilage and synovium in rheumatoid arthritis and osteoarthritis. Osteoarthritis and Cartilage, 9, 42–48. 11178946 10.1053/joca.2000.0348

[cre2588-bib-0010] Blank, V. , Kourilsky, P. , & Israel, A. (1992). NF‐kappa B and related proteins: Rel/dorsal homologies meet ankyrin‐like repeats. Trends in Biochemical Sciences, 17, 135–140. 1533967 10.1016/0968-0004(92)90321-y

[cre2588-bib-0011] Choi, H. , Lee, R. H. , Bazhanov, N. , Oh, J. Y. , & Prockop, D. J. (2011). Anti‐inflammatory protein TSG‐6 secreted by activated MSCs attenuates zymosan‐induced mouse peritonitis by decreasing TLR2/NF‐kappaB signaling in resident macrophages. Blood, 118, 330–338. 21551236 10.1182/blood-2010-12-327353 PMC3138686

[cre2588-bib-0012] Crump, K. E. , Oakley, J. C. , Xia‐Juan, X. , Madu, T. C. , Devaki, S. , Mooney, E. C. , & Sahingur, S. E. (2017). Interplay of toll‐like receptor 9, myeloid cells, and deubiquitinase A20 in periodontal inflammation. Infection and Immunity, 85(1):e00814‐16. 27849177 10.1128/IAI.00814-16 PMC5203663

[cre2588-bib-0013] Danchuk, S. , Ylostalo, J. H. , Hossain, F. , Sorge, R. , Ramsey, A. , Bonvillain, R. W. , Lasky, J. A. , Bunnell, B. A. , Welsh, D. A. , Prockop, D. J. , & Sullivan, D. E. (2011). Human multipotent stromal cells attenuate lipopolysaccharide‐induced acute lung injury in mice via secretion of tumor necrosis factor‐alpha‐induced protein 6. Stem Cell Research & Therapy, 2, 27. 21569482 10.1186/scrt68 PMC3218818

[cre2588-bib-0014] Dang, R. J. , Yang, Y. M. , Zhang, L. , Cui, D. C. , Hong, B. , Li, P. , Lin, Q. , Wang, Y. , Wang, Q. Y. , Xiao, F. , Mao, N. , Wang, C. , Jiang, X. X. , & Wen, N. (2016). A20 plays a critical role in the immunoregulatory function of mesenchymal stem cells. Journal of Cellular and Molecular Medicine, 20, 1550–1560. 27028905 10.1111/jcmm.12849 PMC4956951

[cre2588-bib-0015] Dyer, D. P. , Thomson, J. M. , Hermant, A. , Jowitt, T. A. , Handel, T. M. , Proudfoot, A. E. , Day, A. J. , & Milner, C. M. (2014). TSG‐6 inhibits neutrophil migration via direct interaction with the chemokine CXCL8. Journal of Immunology, 192, 2177–2185. 10.4049/jimmunol.1300194 PMC3988464 24501198

[cre2588-bib-0016] Funk, G. F. , Karnell, L. H. , Robinson, R. A. , Zhen, W. K. , Trask, D. K. , & Hoffman, H. T. (2002). Presentation, treatment, and outcome of oral cavity cancer: A National Cancer Data Base report. Head and Neck, 24, 165–180. 11891947 10.1002/hed.10004

[cre2588-bib-0017] Gilmore, T. D. (2003). The Re1/NF‐kappa B/I kappa B signal transduction pathway and cancer. Cancer Treatment and Research, 115, 241–265. 12613200

[cre2588-bib-0018] Glant, T. T. , Kamath, R. V. , Bardos, T. , Gal, I. , Szanto, S. , Murad, Y. M. , Sandy, J. D. , Mort, J. S. , Roughley, P. J. , & Mikecz, K. (2002). Cartilage‐specific constitutive expression of TSG‐6 protein (product of tumor necrosis factor alpha‐stimulated gene 6) provides a chondroprotective, but not antiinflammatory, effect in antigen‐induced arthritis. Arthtitis and Rheumatism, 46, 2207–2218. 10.1002/art.10555 12209527

[cre2588-bib-0019] Groeger, S. , Hudel, M. , Zechel, S. , Chakraborty, T. , Lochnit, G. , Meyle, J. , & Domann, E. (2021). Generation and functional characterization of recombinant *Porphyromonas gingivalis* W83 FimA. Journal of Biotechnology, 340, 22–29. 34478774 10.1016/j.jbiotec.2021.08.009

[cre2588-bib-0020] Hajishengallis, G. , Darveau, R. P. , & Curtis, M. A. (2012). The keystone‐pathogen hypothesis. Nature Reviews Microbiology, 10, 717–725. 22941505 10.1038/nrmicro2873 PMC3498498

[cre2588-bib-0021] Hayden, M. S. , West, A. P. , & Ghosh, S. (2006). SnapShot: NF‐kappaB signaling pathways. Cell, 127, 1286–1287. 17174900 10.1016/j.cell.2006.12.005

[cre2588-bib-0022] Holt, S. C. , Kesavalu, L. , Walker, S. , & Genco, C. A. (1999). Virulence factors of *Porphyromonas gingivalis* . Periodontology 2000, 20, 168–238. 10522227 10.1111/j.1600-0757.1999.tb00162.x

[cre2588-bib-0023] Hong, J. Y. , Bae, W. J. , Yi, J. K. , Kim, G. T. , & Kim, E. C. (2016). Anti‐inflammatory and anti‐osteoclastogenic effects of zinc finger protein A20 overexpression in human periodontal ligament cells. Journal of Periodontal Research, 51, 529–539. 26548452 10.1111/jre.12332

[cre2588-bib-0024] Huynh, J. , Scholz, G. M. , Aw, J. , Kwa, M. Q. , Achuthan, A. , Hamilton, J. A. , & Reynolds, E. C. (2016). IRF6 regulates the expression of IL‐36 gamma by human oral epithelial cells in response to *Porphyromonas gingivalis* . Journal of Immunology, 196, 2230–2238. 10.4049/jimmunol.1501263 26819203

[cre2588-bib-0025] Inaba, H. , Nakano, K. , Kato, T. , Nomura, R. , Kawai, S. , Kuboniwa, M. , Ishihara, K. , Ooshima, T. , & Amano, A. (2008). Heterogenic virulence and related factors among clinical isolates of *Porphyromonas gingivalis* with type II fimbriae. Oral Microbiology and Immunology, 23, 29–35. 18173795 10.1111/j.1399-302X.2007.00386.x

[cre2588-bib-0026] Ito, T. , Carson, W. F. , Cavassani, K. A. , Connett, J. M. , & Kunkel, S. L. (2011). CCR6 as a mediator of immunity in the lung and gut. Experimental Cell Research, 317, 613–619. 21376174 10.1016/j.yexcr.2010.12.018 PMC3063449

[cre2588-bib-0027] Kota, D. J. , Wiggins, L. L. , Yoon, N. , & Lee, R. H. (2013). TSG‐6 produced by hMSCs delays the onset of autoimmune diabetes by suppressing Th1 development and enhancing tolerogenicity. Diabetes, 62, 2048–2058. 23349496 10.2337/db12-0931 PMC3661629

[cre2588-bib-0028] Kuper, H. , Adami, H. O. , & Trichopoulos, D. (2000). Infections as a major preventable cause of human cancer. Journal of Internal Medicine, 248, 171–183. 10971784 10.1046/j.1365-2796.2000.00742.x

[cre2588-bib-0029] Lamont, R. J. , & Jenkinson, H. F. (1998). Life below the gum line: Pathogenic mechanisms of *Porphyromonas gingivalis* . Microbiology and Molecular Biology Reviews, 62, 1244–1263. 9841671 10.1128/mmbr.62.4.1244-1263.1998 PMC98945

[cre2588-bib-0030] Lee, R. H. , Pulin, A. A. , Seo, M. J. , Kota, D. J. , Ylostalo, J. , Larson, B. L. , Semprun‐Prieto, L. , Delafontaine, P. , & Prockop, D. J. (2009). Intravenous hMSCs improve myocardial infarction in mice because cells embolized in lung are activated to secrete the anti‐inflammatory protein TSG‐6. Cell Stem Cell, 5, 54–63. 19570514 10.1016/j.stem.2009.05.003 PMC4154377

[cre2588-bib-0031] Luster, A. D. (1998). Chemokines–chemotactic cytokines that mediate inflammation. New England Journal of Medicine, 338, 436–445. 9459648 10.1056/NEJM199802123380706

[cre2588-bib-0032] Meng, L. , Zhao, Y. , Bu, W. , Li, X. , Liu, X. , Zhou, D. , Chen, Y. , Zheng, S. , Lin, Q. , Liu, Q. , & Sun, H. (2020). Bone mesenchymal stem cells are recruited via CXCL8‐CXCR2 and promote EMT through TGF‐beta signal pathways in oral squamous carcinoma. Cell Proliferation, 53, e12859. 32588946 10.1111/cpr.12859 PMC7445409

[cre2588-bib-0033] Meyle, J. , Dommisch, H. , Groeger, S. , Giacaman, R. A. , Costalonga, M. , & Herzberg, M. (2017). The innate host response in caries and periodontitis. Journal of Clinical Periodontology, 44, 1215–1225. 28727164 10.1111/jcpe.12781

[cre2588-bib-0034] Milner, C. M. , Higman, V. A. , & Day, A. J. (2006). TSG‐6: A pluripotent inflammatory mediator? Biochemical Society Transactions, 34, 446–450. 16709183 10.1042/BST0340446

[cre2588-bib-0035] Milward, M. R. , Chapple, I. L. , Wright, H. J. , Millard, J. L. , Matthews, J. B. , & Cooper, P. R. (2007). Differential activation of NF‐kappaB and gene expression in oral epithelial cells by periodontal pathogens. Clinical and Experimental Immunology, 148, 307–324. 17355248 10.1111/j.1365-2249.2007.03342.x PMC1868880

[cre2588-bib-0036] Mindrescu, C. , Dias, A. A. , Olszewski, R. J. , Klein, M. J. , Reis, L. F. , & Wisniewski, H. G. (2002). Reduced susceptibility to collagen‐induced arthritis in DBA/1J mice expressing the TSG‐6 transgene. Arthtitis and Rheumatism, 46, 2453–2464. 10.1002/art.10503 12355494

[cre2588-bib-0037] Muir, C. , & Weiland, L. (1995). Upper aerodigestive tract cancers. Cancer, 75, 147–153. 8000993 10.1002/1097-0142(19950101)75:1+<147::aid-cncr2820751304>3.0.co;2-u

[cre2588-bib-0038] Nakagawa, I. , Amano, A. , Kuboniwa, M. , Nakamura, T. , Kawabata, S. , & Hamada, S. (2002). Functional differences among FimA variants of *Porphyromonas gingivalis* and their effects on adhesion to and invasion of human epithelial cells. Infection and Immunity, 70, 277–285. 11748193 10.1128/IAI.70.1.277-285.2002 PMC127611

[cre2588-bib-0039] Nakano, K. , Inaba, H. , Nomura, R. , Nemoto, H. , Takeuchi, H. , Yoshioka, H. , Toda, K. , Taniguchi, K. , Amano, A. , & Ooshima, T. (2008). Distribution of *Porphyromonas gingivalis* fimA genotypes in cardiovascular specimens from Japanese patients. Oral Microbiology and Immunology, 23, 170–172. 18279186 10.1111/j.1399-302X.2007.00406.x

[cre2588-bib-0040] Oltulu, Y. M. , Coskunpinar, E. , Ozkan, G. , Aynaci, E. , Yildiz, P. , Isbir, T. , & Yaylim, I. (2014). Investigation of NF‐kappaB1 and NF‐kappaBIA gene polymorphism in non‐small cell lung cancer. BioMed Research International, 2014, 530381. 24707489 10.1155/2014/530381 PMC3953471

[cre2588-bib-0041] Parkin, D. M. , Bray, F. , Ferlay, J. , & Pisani, P. (2005). Global cancer statistics, 2002. CA: A Cancer Journal for Clinicians, 55, 74–108. 15761078 10.3322/canjclin.55.2.74

[cre2588-bib-0042] Proudfoot, A. E. (2002). Chemokine receptors: Multifaceted therapeutic targets. Nature Reviews Immunology, 2, 106–115. 10.1038/nri722 PMC7097668 11910892

[cre2588-bib-0043] Qi, Y. , Jiang, D. , Sindrilaru, A. , Stegemann, A. , Schatz, S. , Treiber, N. , Rojewski, M. , Schrezenmeier, H. , Vander Beken, S. , Wlaschek, M. , Bohm, M. , Seitz, A. , Scholz, N. , Durselen, L. , Brinckmann, J. , Ignatius, A. , & Scharffetter‐Kochanek, K. (2014). TSG‐6 released from intradermally injected mesenchymal stem cells accelerates wound healing and reduces tissue fibrosis in murine full‐thickness skin wounds. Journal of Investigative Dermatology, 134, 526–537. 23921952 10.1038/jid.2013.328

[cre2588-bib-0044] Ranasinghe, R. , & Eri, R. (2018). Pleiotropic immune functions of chemokine receptor 6 in health and disease. Medicines, 5(3), 69. 10.3390/medicines5030069 PMC6164274 30004409

[cre2588-bib-0045] Reid, B. C. , Winn, D. M. , Morse, D. E. , & Pendrys, D. G. (2000). Head and neck in situ carcinoma: Incidence, trends, and survival. Oral Oncology, 36, 414–420. 10964047 10.1016/s1368-8375(00)00028-2

[cre2588-bib-0046] Sallusto, F. , & Baggiolini, M. (2008). Chemokines and leukocyte traffic. Nature Immunology, 9, 949–952. 18711431 10.1038/ni.f.214

[cre2588-bib-0047] Sbordone, L. , & Bortolaia, C. (2003). Oral microbial biofilms and plaque‐related diseases: Microbial communities and their role in the shift from oral health to disease. Clinical Oral Investigations, 7, 181–188. 14598129 10.1007/s00784-003-0236-1

[cre2588-bib-0048] Schutyser, E. , Struyf, S. , & Van Damme, J. (2003). The CC chemokine CCL20 and its receptor CCR6. Cytokine and Growth Factor Reviews, 14, 409–426. 12948524 10.1016/s1359-6101(03)00049-2

[cre2588-bib-0049] Socransky, S. S. , & Haffajee, A. D. (2002). Dental biofilms: Difficult therapeutic targets. Periodontology 2000, 28, 12–55. 12013340 10.1034/j.1600-0757.2002.280102.x

[cre2588-bib-0050] Song, S. , Chen, D. , Lu, J. , Liao, J. , Luo, Y. , Yang, Z. , Fu, X. , Fan, X. , Wei, Y. , Yang, L. , Wang, L. , & Wang, J. (2011). NFkappaB1 and NFkappaBIA polymorphisms are associated with increased risk for sporadic colorectal cancer in a southern Chinese population. PLoS One, 6, e21726. 21738780 10.1371/journal.pone.0021726 PMC3128094

[cre2588-bib-0051] Souto, G. R. , Queiroz, C. M. Jr. , Costa, F. O. , & Mesquita, R. A. (2014). Relationship between chemokines and dendritic cells in human chronic periodontitis. Journal of Periodontology, 85, 1416–1423. 24605873 10.1902/jop.2014.130662

[cre2588-bib-0052] Szanto, S. , Bardos, T. , Gal, I. , Glant, T. T. , & Mikecz, K. (2004). Enhanced neutrophil extravasation and rapid progression of proteoglycan‐induced arthritis in TSG‐6‐knockout mice. Arthtitis and Rheumatism, 50, 3012–3022. 10.1002/art.20655 15457471

[cre2588-bib-0053] Tezal, M. , Sullivan, M. A. , Hyland, A. , Marshall, J. R. , Stoler, D. , Reid, M. E. , Loree, T. R. , Rigual, N. R. , Merzianu, M. , Hauck, L. , Lillis, C. , Wactawski‐Wende, J. , & Scannapieco, F. A. (2009). Chronic periodontitis and the incidence of head and neck squamous cell carcinoma. Cancer Epidemiology, Biomarkers and Prevention, 18, 2406–2412. 10.1158/1055-9965.EPI-09-0334 19745222

[cre2588-bib-0054] Tezal, M. , Sullivan, M. A. , Reid, M. E. , Marshall, J. R. , Hyland, A. , Loree, T. , Lillis, C. , Hauck, L. , Wactawski‐Wende, J. , & Scannapieco, F. A. (2007). Chronic periodontitis and the risk of tongue cancer. Archives of Otolaryngology—Head and Neck Surgery, 133, 450–454. 17515503 10.1001/archotol.133.5.450

[cre2588-bib-0055] Wilhelm, B. T. , Marguerat, S. , Watt, S. , Schubert, F. , Wood, V. , Goodhead, I. , Penkett, C. J. , Rogers, J. , & Bahler, J. (2008). Dynamic repertoire of a eukaryotic transcriptome surveyed at single‐nucleotide resolution. Nature, 453, 1239–1243. 18488015 10.1038/nature07002

[cre2588-bib-0056] Wisniewski, H. G. , Maier, R. , Lotz, M. , Lee, S. , Klampfer, L. , Lee, T. H. , & Vilcek, J. (1993). TSG‐6: A TNF‐, IL‐1‐, and LPS‐inducible secreted glycoprotein associated with arthritis. Journal of Immunology, 151, 6593–6601. 8245487

